# Place Cells, Grid Cells, Attractors, and Remapping

**DOI:** 10.1155/2011/182602

**Published:** 2011-11-03

**Authors:** Kathryn J. Jeffery

**Affiliations:** Department of Cognitive, Perceptual and Brain Sciences, University College London, 26 Bedford Way, London WC1H 0AP, UK

## Abstract

Place and grid cells are thought to use a mixture of external sensory information and internal attractor dynamics to organize their activity. Attractor dynamics may explain both why neurons react coherently following sufficiently large changes to the environment (discrete attractors) and how firing patterns move smoothly from one representation to the next as an animal moves through space (continuous attractors). However, some features of place cell behavior, such as the sometimes independent responsiveness of place cells to environmental change (called “remapping”), seem hard to reconcile with attractor dynamics. This paper suggests that the explanation may be found in an anatomical separation of the two attractor systems coupled with a dynamic contextual modulation of the connection matrix between the two systems, with new learning being back-propagated into the matrix. Such a scheme could explain how place cells sometimes behave coherently and sometimes independently.

## 1. Introduction

**Figure 1:**
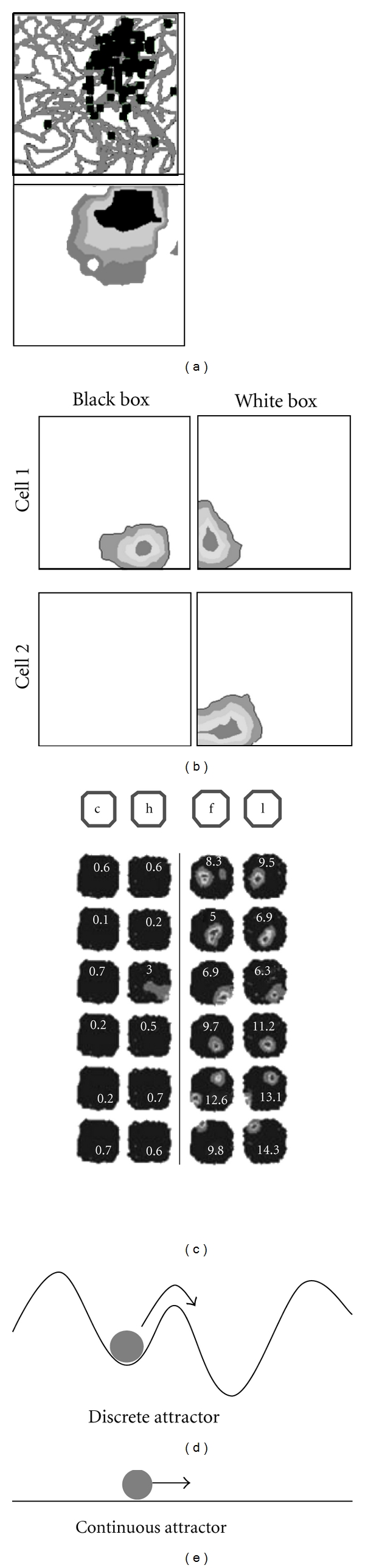
(a) Activity of a CA1 place cell, recorded as a rat foraged for rice grains in a 60 cm-square box for four min. The top plot shows the raw spikes (black squares) superimposed on the path of the rat as it (grey line), and the bottom plot shows a contour plot of firing rate, shown in 20% gradations (black = the peak 20% region). (b) Examples of remapping from two cells in CA1, as a box was changed from black to white. Cell 1 remapped by changing the location of its firing field, while Cell 2 remapped by switching its field off. (c) Evidence for discrete attractors in CA1, adapted from [9]. As an environment was incrementally “morphed” between more circle-like and more square-like (visible as the slightly lengthening corners as the diagrams go from left to right), the cells abruptly and coherently (all together) changed their activity. (d) Cross-sectional attractor landscape for a discrete attractor. The stable states of the system are the hollows into which the system (analogous to a rolling ball) settles and from which it resists perturbation. (e) A continuous attractor which does not resist perturbations but moves smoothly from one state to an adjacent one.

The hippocampal place cells are thought to collectively form a representation of space, known as a “cognitive map” [1], because of their spatially localized firing, which occurs in patches known as place fields (Figure 1(a)). One source of spatial inputs to place cells is the entorhinal grid cells, one synapse upstream, whose activity forms a regular array of firing fields [2] suggestive of an intrinsic odometric (distance-measuring) process, which may convey metric information to place cells and allow them to position their place fields accurately in space [3]. The place and grid cells are an excellent model system with which to study the formation and architecture of cognitive knowledge structures.

Place and grid cells use external environmental cues to anchor their activity to the real world, as evidenced by the fact that their activity appears bound to the local environmental walls [2, 4, 5] and reacts to changes in the environment [6]. However, firing patterns are then stabilized and maintained by internal network dynamics so that activity can be self-sustaining and coherent across the network. These internal dynamics are often considered to arise from the operation of attractor processes [7–9], which are processes that arise from mutually interconnected neurons that collectively have a tendency to find stable states. Two kinds of attractors have been proposed to explain place cell behavior: discrete and continuous. The purpose of this paper is to review the evidence for these two attractor types in the hippocampal network and then to explore a phenomenon that cannot be easily accounted for by attractors, known as partial remapping. Finally, a model will be described that may be able to explain how both attractor dynamics and partial remapping can co-exist in the same network.

### 1.1. Attractors and Place Cell Remapping

One of the earliest and most striking observations concerning the place cell representation was the way that the cells can suddenly and collectively alter their activity from one pattern to another, a process known as remapping ([6] Figure 1(b)). This phenomenon led to proposals that the pattern of activity arises from cooperative activity among all involved place neurons, perhaps exerted via the recurrent synapses in the highly interconnected CA3 network [8]. The attractor hypothesis built upon earlier ideas that the hippocampal CA3 network functions as an autoassociative memory [10–12]. Attractor networks are a special case of autoassociative memory, and an attractor's defining characteristic is the existence of stable states, caused by the mutual excitation of neurons within the network, towards which the system gravitates when it is sufficiently close. 

The process of moving towards and settling into a stable state is what is meant by “attractor dynamics”. Anatomical and physiological observations of place cells suggest the operation of two kinds of attractor dynamics: discrete and continuous. Discrete attractor dynamics enable the system to resist small changes in sensory input but respond collectively and coherently to large ones, while continuous dynamics enable the system to move smoothly from one state to the next as the animal moves through space [7]. These two attractor systems clearly must either be colocalized on the same neurons or else be separate but interacting, since one accounts for the population of place cells active at a given moment and the other for the progression of activity from one set to the next as the animal moves. One possibility, discussed later, is that the source of the discrete attractor dynamics may lie in the place cell network itself [7–9, 14], and the continuous dynamics may originate upstream in the entorhinal grid cell network [15]. 

In a discrete attractor network, the possible states are clearly separable, and when the system moves from one state to another, it seems to do so abruptly. The separate states of a discrete attractor are often conceptualized as hollows in an undulating energy landscape (Figure 1(d)) into which the system (represented as a ball) tends to gravitate (i.e., to be attracted to). The hollows, also called basins, are low-energy states, but to move from one hollow to the next, the ball requires a substantial perturbation: a small push will not cause it to change basins/states. The states are imprinted onto the network by appropriate modulation of the connection strengths between the neurons in the network [16]. Place cell remapping was initially conceptualized as being a sudden transition of the place cell network from one state to the next following the large perturbation arising from environmental change [17], an idea that has been very influential.

Experimental evidence for attractor dynamics in the place cell network was initially provided by observations of remapping, but stronger evidence came from a study by Wills et al. [9], who showed that incremental changes in the squareness or circularity of an enclosure would produce no change in place cell activity until the cumulative changes became sufficiently great, at which point the whole system would suddenly switch to the other pattern (Figure 1(c)). Interestingly, attractor dynamics do not seem to be invariable observations: for example, they were not seen by Leutgeb et al. [18], who found, by contrast, that gradual transformation of environment shape induced gradual transition of firing patterns from one shape to the other. Such differences in network behavior may be accounted for by the “attractor landscape”—the exact distribution of the imaginary hills and valleys in the attractor state-space—which may have been sculpted by the past experience of the animal, via processes described below.

Attractor properties derive from the pattern of recurrent synaptic connections, and attractor networks can learn new information, presumably by Hebbian synaptic plasticity occurring in these connections [16]. In support of this notion, the place cell network is highly plastic, as evidenced by the propensity for its synapses to display changes in strength, either upwards (long-term potentiation; LTP) or downwards (long-term depression; LTD), in response to activity patterns in its afferents [19]. Exactly what this plasticity is for is still unresolved, but one hypothesis has been that one function is for the system to discover for itself the different states the environment can take and to represent this by different states (also called “charts” [7]) in the discrete attractor landscape [14]. Place cells do indeed have the capacity to acquire different representations of an environment that was previously represented by a single state [20, 21], an observation which is consistent with this idea. 

The attractor architecture allows for comparison of incoming sensory information with stored information in the network. The incoming information effectively places the attractor network in a state that is nearer to or further from an attractor basin. When presented with an altered state of an environment (e.g., an office with the furniture rearranged or a familiar field covered in snow), the system compares its stored representations with the current observed state and makes a decision about whether the current state is the same environment after minor changes (pattern completion), or a different environment requiring a new representation (pattern separation). The decision comprises movement of the system to the nearest stable state. Whether or not the system opts to separate (move to an adjacent basin) or complete (return to the previous basin) the pattern, the cells should all act coherently by virtue of the attractor architecture. Experimental evidence for pattern completion in the CA3 hippocampal network was provided by Nakazawa et al. [22] who found that partial presentation of a set of environmental cues (analogous to placing a ball at the edge rather than the centre of an attractor basin) triggered spontaneous retrieval of the full activation pattern. 

The other property characteristic of place cells is the way that a given activity pattern can smoothly move from one to the next as an animal moves through space. This smooth movement has also been ascribed to attractor dynamics in a recurrent network, but instead of a discrete attractor in which the state jumps from one pattern to the next, it is thought instead to comprise a “continuous attractor” around which the activity moves smoothly [7, 17]. A continuous attractor can be conceptualized as movement of the imaginary ball across a smooth surface rather than a hilly landscape (Figure 1(e)). The “attractors” in this network are no longer the possible states of the network in the whole environment, but rather the activity patterns that pertain across the active cells when the animal is at one single place in that environment [17]—any neuron that is supposed to be part of this state, at that place, will tend to be pulled into it and held there by the activity of the others to which it is connected. Continuous attractors were originally postulated in order to explain the dynamics of the upstream head direction cells [23]. For place cells, the one-dimensional “ring” attractor of the head direction cell model has been extended to two dimensions [7]. 

### 1.2. Partial Remapping: A Challenge for Attractor Hypotheses

One problem with the notion of attractors in the hippocampal network is that under some situations, place cells fail to act coherently. This phenomenon, which is known as partial remapping [24], occurs when an environmental change causes some cells to remap and others to maintain their firing patterns unaltered. It is frequently observed when partial changes are made to an environment such that some cues change while others do not [13, 24–26]. Partial remapping is rarely addressed in models of remapping, perhaps because it is not seen in the more typical experimental paradigms involving large environmental changes and also perhaps because of the theoretical difficulties it introduces. Partial remapping is difficult for an attractor model to explain [27], because the defining feature of attractors is the coherence of the network behavior and partial remapping represents a degree of *in*coherence. 

**Figure 2:**
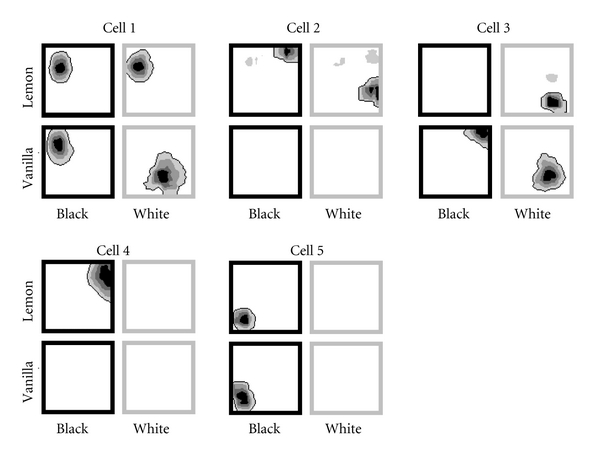
Fragmentation of attractors. The figure shows the firing fields of five simultaneously recorded cells from the experiments described in [13] that were recorded as rats were exposed to four different contexts comprising pairwise combinations of a color (black or white) and an odor (lemon or vanilla). The most straightforward version of the attractor hypothesis would predict that when remapping occurs, all cells should remap together. However, it can be seen here that in the lemon box (top panels), when the color was changed from black to white, then cell 1 did not remap while the remaining cells did—cell 2 by shifting its field, cell 3 by switching on a field, and cells 4 and 5 by switching off their fields. When the box was vanilla, however, then cells 1 and 3 responded by remapping, cells 2 and 4 did not fire at all in either vanilla condition, and cell 5 switched off its field. Thus, each cell seemed to act independently and not as part of an attractor, bound to the others. Furthermore, each cell reacted to a *combination* of color and odor—the response to odor change was conditional upon what the color of the box was, and vice versa.

It is possible to circumvent this problem by supposing that under some situations attractors can fragment into submaps (or “maplets” [28]) so that some of the neurons (the ones that did not remap, say) belong to one attractor system and the others (the ones that did remap) to a second one. However, in a study of bidimensional contextual remapping [13], we found that the attractor could break down even further. Here, contextual stimuli were varied across two stimulus dimensions, color and odor, and cells were found to respond essentially arbitrarily to the different combinations. This is shown in the examples in Figure 2, in which the recording environment, a square box, could be varied in either color (black or white) and odor (lemon or vanilla). The cells in this example have clearly responded as individuals to these changes. Even with these limited environmental changes, the five cells shown here would require five attractor maplets, since no two cells have remapped in quite the same way. Once it becomes necessary to propose as many attractors subsystems as there are neurons, the attractor concept starts to lose its explanatory power.

One way to rescue the attractor hypothesis is to suppose that perhaps attractors are normally created in the place cell system, but our experiment created a pathological situation in which the ability of the network to discover attractor states was thwarted by the way in which context elements were explicitly decorrelated so that no two always occurred together. Perhaps, partial remapping is a reflection of a broken attractor system—one in which the neurons act independently because their ability to act cohesively was disrupted by the fragmented nature of the environments we created. An alternative possibility is that partial remapping is a normal reflection of simultaneous encoding of both similarities and differences in two contexts [29]. However, regardless of whether partial remapping reflects a pathology in the discrete attractor network in the hippocampus or is a normal reflection of configural encoding, its existence poses an interesting conundrum, because although the discrete attractors appear to have fragmented, the continuous attractor dynamics seem intact: activity can still move smoothly from one set of neurons to the next even though some of the neurons seem to belong to one subnetwork and some to another, and partial remapping to a new environmental configuration may have thrown up a combination of place fields that has never occurred before. It seems that fragmentation of the discrete attractor networks has not interrupted the continuous attractor dynamics. The discussion below reviews evidence that this may be because the continuous and discrete attractors are in different networks, with the continuous attractor dynamics residing in the network in entorhinal cortex [15] and the discrete attractor landscape resulting from plasticity processes in the dentate-CA3 network in hippocampus. The discrete dynamics—the shift of a given place cell from one pattern to another—will be explained as the result of contextually modulated switching in the entorhinal-hippocampal connections.

### 1.3. The Grid Cells

The entorhinal grid cells, upstream of the place cells in dorsomedial entorhinal cortex, were discovered by Hafting et al. in the Moser lab [2]. The firing of grid cells is spatially localized, but firing fields from a given cell are multiple and occur in evenly spaced arrays of circular fields arranged in a hexagonal close-packed configuration (which happens to be the most efficient way of tiling a plane with circles). Grid cells have a number of interesting features which make them plausible candidates for the long-postulated continuous attractor system that underlies place cell activity and the ability of place cells to update their activity in response to movement [31]. To begin with, they are always active in any environment, like head direction cells but unlike place cells. Also, as far as we know, closely colocalized (and possibly also more distant) cells having the same grid scale maintain the same relative firing field locations, regardless of the absolute location with respect to the world outside. This suggests the operation of intrinsic network dynamics, in which activity is modulated by movement of the animal in any direction but reinforced by intrinsic connections in the grid cell network itself.

How is this network activity conveyed to place cells? The spatial nature of grid cell firing makes these cells natural candidates to underlie place field formation, but the issue of how the grid cell activity patterns are converted into place fields is not yet resolved. Evidence suggests that there is a high convergence from grid cells to dentate granule cells, CA3 cells, and CA1 cells: for example, de Almeida et al. estimated that each granule cell receives around 1200 synapses from grid cells [32]. Importantly, grid fields occur at different spacings at different dorsoventral levels, and a place cell receives inputs from a variety of different scales [33]. However, modeling shows that even when multiple grid scales converge, this number of grids impinging on a single neuron results in a cell being activated uniformly across the whole environment. Even when the gain of the inputs is turned down so that the place cell only becomes active at the peaks of this drive, the resulting activity hotspots occur in a highly regular, multipeaked symmetrical array that does not resemble real place fields [34]. One way around this problem is to produce some kind of inhomogeneity or chunking of the grid cell inputs so that the resulting pattern becomes lumpy and more likely to produce a small number of fields [32]. This can be done by increasing the sparseness of the network [35], varying orientation and spatial phase [34], adding phase precession [36], grouping the inputs according to spatial phase [37], or adding feedback inhibition and varying synaptic strengths [32]. The resulting patterns have something of a resemblance to dentate granule cell place fields, which are multipeaked, like grid fields, but irregular like place fields, and intermediate in sparsity between grid and place fields [38]. Such schemes do not, however, account for some features of place cell activity, such as why fields tend to be elongated near walls [39], and so clearly, some additional factor is needed to fully account for place field morphology. This will not be addressed further here, however.

Having established a basic possible connectivity between grid and place cells and also that the grid cells are the likeliest source of the continuous attractor dynamics in the system, the question now is how the continuous attractor dynamics of the place cells can be explained as the animal moves around. This is relatively straightforward: because the activity of place cells derives from the grids, then as the grid cell activity rises and falls with locomotion, so too should the drive onto the place cells, with the irregularity resulting from a combination of the multiple scales and (perhaps) the chunked inputs. Because the place cells inherit the attractor dynamics from the grid cells and not from each other, it therefore does not matter if the place cell network is disrupted by partial remapping.

### 1.4. “Remapping” in Grid Cells

**Figure 3:**
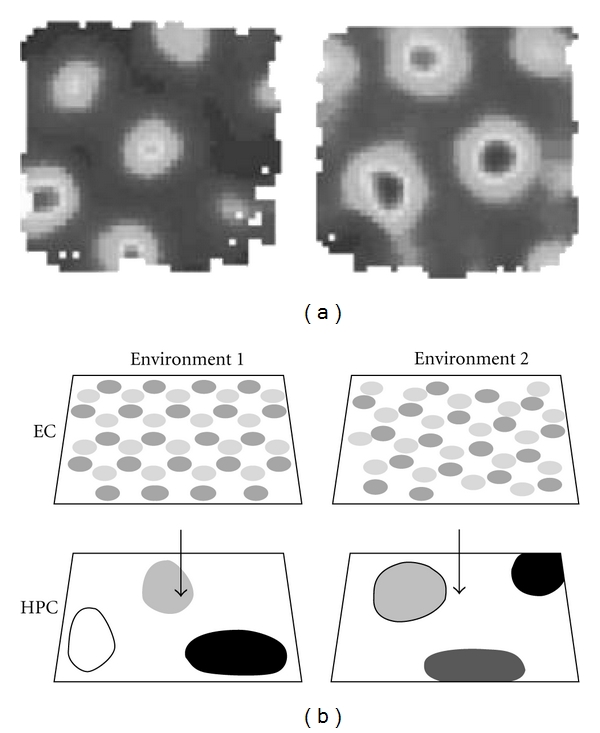
(a) Data adapted from Fyhn et al. [30] showing how grid fields remap (translate and rotate) following movement of the rat from one room to another. (b) Schematic illustration of remapping following environmental change, in grid cells and place cells. EC: entorhinal cortex, HPC: hippocampus. Sets of grid cells (represented here by two offset grid arrays, with one grid dark grey and the other light grey) project to place cells and generate a set of place fields. When the environment changes, the grid cells remap (in this case, with a translation and a rotation). Note that the two grid cells, although they have altered their absolute firing positions, have maintained the same relative positions with respect to each other. The place cells also remap, with, in this example, two cells switching fields off, two switching them on and one shifting its field to a new location in the enclosure.

Like place cells, grid cells change their firing patterns following environmental change [2, 30], but the exact nature of the remapping is different. As mentioned above, grid cells are always active, so they do not switch fields on and off as place cells do. Nor do they, as far as is known, modulate their rates in a rate-remapping fashion, like place cells do in some situations [21, 40]. Rather, their “remapping” consists of translation and/or rotation of fields (Figure 3). In the first systematic study of grid cell remapping by Fyhn et al. [30], large changes to the environment, such as moving the animal to a new room, caused both translation and rotation, while small changes (changing the enclosure but not the room) did not cause remapping at all. Interestingly, the large changes were accompanied by place cell “global” remapping (reshuffling of the map), while small changes were associated only with rate remapping. Rate remapping occurs when the response of a place cell to a change in context is to increase or decrease the intensity of the place field without switching on or off completely [21, 40].

Following environmental change, the grid cells seem to act coherently, inasmuch as any sufficiently large change seems to cause the firing fields to shift *en masse* [2, 30]. This poses some problems for models of place field generation: if the grid cells are, as popularly supposed, the basis for place field generation, then how can the heterogeneity of place field remapping be accounted for by the apparent homogeneity of grid cell remapping?

Place field heterogeneity takes two forms. The first is that during complete remapping, some cells switch their fields on or off and some cells shift their fields (Figure 3(b)). The resulting pattern has, as far as we can determine, no relationship to the original and the map looks to have been randomly regenerated. Thus, the fields alter their position, or their very existence, with respect to each other. Since attractor dynamics arise from the connections between the neurons in the map, this means the original attractor, if it were located in the intrahippocampal connections, must have been disrupted. As discussed above, the solution to this problem may be that the continuous attractor dynamics reside in the grid cell network and not the place cell network. 

While this could explain the continuity of continuous attractor dynamics across a change in environment, it still does not explain the place field response of rearrangement of fields, because mere rotation and translation of a grid array should cause simple rotation and translation of the place field array, which clearly does not happen. Fyhn et al. [30] suggested two ways around this problem: first, perhaps there is modularity in the grid cell population such that the whole population does shift, or second, perhaps the grid pattern shifts to a point far away on an imaginary, infinite grid field array, which would result in the place fields effectively shifting and rotating by such a large amount that the resulting place field pattern is one that did not previously occur in the enclosure. We have put forward an alternative explanation, outlined in the next section.

The other kind of heterogeneity is the partial remapping discussed above, where some cells respond to a particular environmental change, while others do not (Figure 2). This seems harder to explain by grids, because if the grid cells are the drivers of place cell activity, then how can partial remapping occur? Do grid cells remap partially? There are no published data to support this yet, but evidence so far suggests that the grid cells mostly tend to act coherently [2, 30]. It may be, however, that grids are not really as homogeneous as has been assumed and that they can in fact remap independently, and thus become dislocated with respect to each other. This seems unlikely however. 

The other, perhaps more plausible possibility is that the mapping between the grid cells and place cells can be dynamically modulated so that following an environmental change, the population of grid cells that drives a particular place cell becomes altered. We have previously proposed that such modulation could occur by means of concurrently active contextual inputs-the inputs that tell the system that the environment has changed [13, 37, 41, 42]. This model is described, below, and an outline provided of how it may explain how the homogeneous and always-present pattern of grid cell activity can translate to the heterogeneous and sometimes-present activity of place cells.

## 2. The Contextual Gating Model

**Figure 4:**
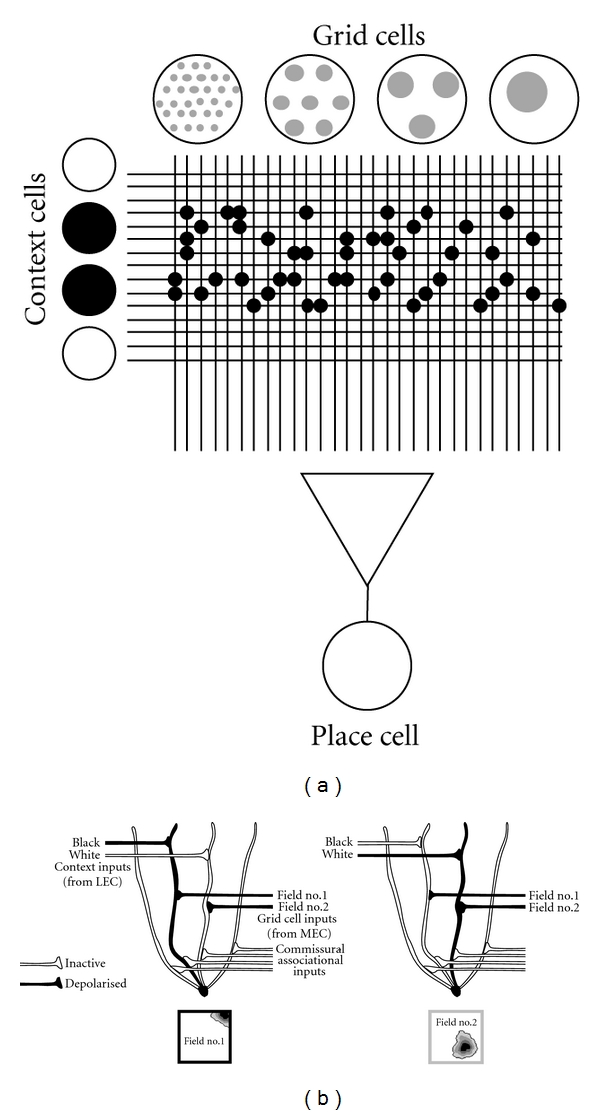
The contextual gating model of place field generation. (a) Place cells receive inputs from grid cells of varying scales (shown by the small versus large circles in the units). The connections are intercepted by convergent contextual inputs which facilitate (or perhaps inhibit) the grid cell inputs and thus determine which grid cells actually drive the place field. In this way, constant activity from the grid cells is converted into context-specific activity in the place cells. When the context is changed, the matrix of activated synaptic connections alters—if this is sufficiently large then a new pattern of grid cells will drive the place cell to fire differently (completely remap); if it is small, the cell will still fire in the same place, but perhaps with a different rate (rate remap). (b) A schematic of the context gating model at the single-cell level, showing how the contextual inputs in the distal dendrites terminate on the same branch of the dendritic tree as some of the spatial (grid cell) inputs. In the black box (left), the context inputs signalling that the box is black interact synergistically with a subset of the spatial inputs, producing the field seen in the black box below; when the box is white (right), a different branch of the dendrite is depolarised and a different set of spatial inputs facilitated.

In 2008 we presented a “contextual gating” model of grid-cell/place-cell connectivity that may explain how the heterogeneous and seemingly individualistic behavior of place cells might arise from the relatively homogeneous and coherent activity of grid cells [37].  Figure 4 illustrates the basic model, comprising a set of grid cells projecting to place cells in hippocampus in order to drive the formation of place fields. Converging onto these same cells are a set of inputs conveying information about context, such as whether the box is black/white or lemon/vanilla as in the example given earlier. The function of these context inputs is to interact with the spatial inputs from the grid cells and decide which of the spatial inputs “get through” and can drive the granule cell.  Figure 4(a) shows a schematic of the synaptic matrix arising from the intersection of the context inputs and spatial inputs. This matrix transforms the uniform and coherent pattern of grid cell activity into the discrete and cell-specific patterns seen in dentate gyrus. Thus, the contextual inputs have “gated” the spatial inputs. 

Figure 4(b) shows the model at the single neuron level. The figure shows a hypothetical hippocampal neuron, in this case a dentate granule cell, which integrates convergent spatial and contextual information in its dendrites. The schematic illustrates how inputs from medial and lateral entorhinal cortex converge, in the dentate gyrus, onto the same set of granule cells, with the grid cell inputs arriving from medial entorhinal cortex (MEC) and terminating on the middle portion of the dendritic tree, and inputs carrying contextual information arriving from the lateral entorhinal cortex (LEC) and terminating in the outer part of the dendritic tree. (There are also feedback inputs from the dentate-CA3 network arriving via the commissural-associational network, which terminate in the proximal dendrites, as discussed later.) In our model, the basic unit of computation is a branch of the dendritic tree, which receives both spatial and contextual inputs and is able to integrate these. Interestingly, the idea that a dendritic branch could be a unit of processing is one that is gaining increasing support in the experimental literature [43].

The figure illustrates how, using this model, complete remapping can be explained as follows: when the context changes, now a different set of context inputs is active and these gate a different set of spatial inputs, driving a different branch of the granule cell dendritic tree, and thus generating a different spatial pattern of activity. If the change to the environment is large, then there is a massive change in the pattern of context inputs which would affect all the grid cell inputs to all the place cells, causing a complete remapping. For smaller changes, some of the inputs to the place cell would alter, and some would stay the same—depending on how big these changes were the cell might retain its original place field. Similarly, rate remapping can be explained by a change of the facilitation level of the context inputs to the grid cell ones. This change could be either up or down, consistent with real data. Importantly, different cells are able to be modulated independently, also consistent with real data [38].

The most important feature of the contextual gating model is that it can explain partial remapping. Even if activity in the grid cell sheet continues at the same level (with or without translation/rotation remapping), and with the same spatial relationship between the field arrays of individual cells, the model allows for independent tuning of individual cells, meaning that an environmental change is able to affect some cells but not others. 

We have modeled this contextual gating proposal [37] by simulating networks of grids of varying scales which project to granule cells, and then altering the connection to each granule cell in a context-dependent manner. The model possesses a number of important features. First, in order to generate realistic-looking dentate granule fields, which could, in turn, produce realistic CA3 fields, we needed to introduce some kind of chunking of the inputs, for reasons discussed earlier. We accomplished this by grouping the inputs according to spatial phase (“offset”) so that grid cells having overlapping grid fields would be more likely to coterminate on a particular branch of a granule cell dendrite, in “offset clusters”. Each dendritic branch, possessing grid cell inputs with an above-average tendency to overlap in a region of the environment, formed the computational unit of the network. The clustering of the grid cell inputs in this way helped to avoid uniform activation across the environment, and with appropriate choice of grouping parameter, we were able to produce granule cell activation that occurred in only a few places in the environment, consistent with the multipeaked irregular place fields in the data from Leutgeb et al. [38].

**Figure 5:**
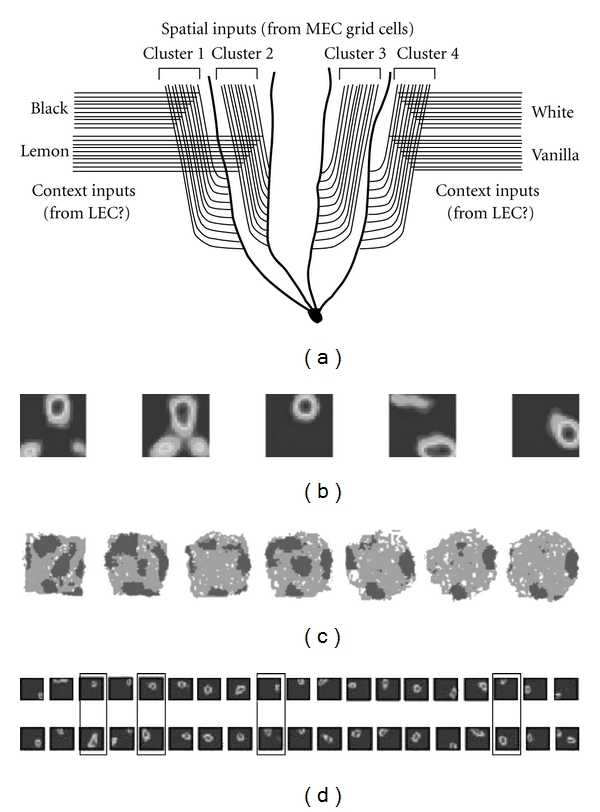
(a) Schematic of the proposed clustering of grid cell inputs to a granule cell dendritic tree, together with convergence of clusters of contextual inputs (in this case, the context elements from the experiments described in [13]). Some of the context elements (in this case, white and vanilla) coterminate on the same dendritic branch, and thus gate the same set of grid cells, accounting for the configural behaviour of place cells that are able to respond by, for example, generating a different place field for white-vanilla than for any of the other environments, like cell 1 in Figure 2. ((b)—(d)) Dentate gyrus subfield remapping, obtained both via modelling ((b); [37]) and from experimental data ((c); [38]). The modelling data in (b) reflect the effects of progressively changing the contextual inputs that facilitate the drive from grid cells to place cells and reveal that this has the effect of progressively shifting activation from some subfields to others. The plots in (c) show that this effect is also seen in the real data. (d) Modelling data showing production of partial remapping in simulated CA3 neurons following the contextual dentate remapping shown in (b). Cells whose fields shifted location are shown by the rectangular outlines.

Next, we introduced contextual modulation of the kind discussed above so that a given cluster of entorhinal inputs—that is, those terminating on one branch of the cell's dendritic tree—could only drive the granule cell if the appropriate contextual inputs were also active and terminating on that same dendritic branch. By switching context inputs (and hence dendritic branches and their associated inputs) on and off, we mimicked the effect of placing a rat in different environments. By steadily (rather than abruptly) varying the degree of contextual variation, we were able to cause the simulated granule cells to progressively change their firing patterns (Figure 5). Interestingly, both the model data from our simulation and the real data from Leutgeb et al. show the same effect, which is that rather than producing on-off remapping as is typical with place cells, we saw a gradual refocusing of the subfields of a set of granule cells. Thus, as the context was progressively altered (by varying the contextual drive in the simulation, or by slowly “morphing” the environment in the real experiment), it was possible to slowly shift activity from one subfield to another within the granule cell field cluster. A similar effect has subsequently also been reported by Rennó-Costa et al. who explored the effect quantitatively [44]. These gradual changes translated into place field remapping in a simulated downstream CA3 network (Figure 5(c)).

Our model can explain other features of place cell activity. One example is rate remapping, which could be accounted for in the contextual gating model by supposing that rather than altering the contextual inputs completely, and switching in a new set of grids, the environmental change merely decreases the intensity of the inputs coming in, and thus weakens the synergistic interaction between the contextual and spatial inputs.

The model can also explain partial remapping in the place cells, downstream from the granule cells. By generating simulated CA3 fields from the granule cells, we found a predominance of unitary fields, which closely resemble real place fields (although, as mentioned earlier, they lack some notable features such as conformation to wall contours). By changing the contextual inputs, we showed that some CA3 fields did not change while others remapped (Figure 5(c)).

### 2.1. Conditional Remapping

**Figure 6:**
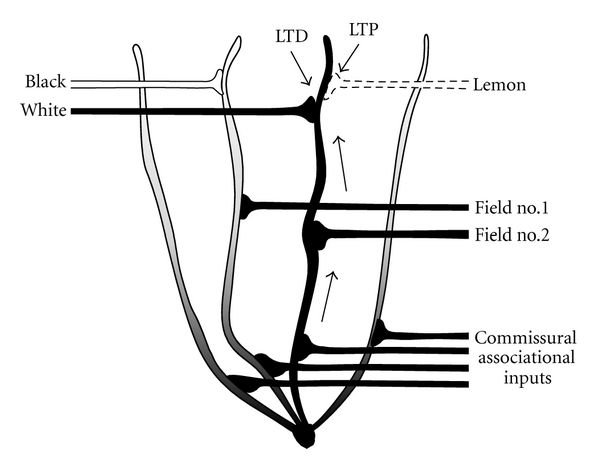
Extension of the context gating model to account for the learning of new combinations of contextual inputs and new attractor states. As well as the contextual and spatial inputs shown in Figure 4, there are also feedback connections from the commissural associational fibre system, which retrogradely depolarise the dendritic tree (shading). In those dendritic branches that also are receiving anterograde contextual depolarisation (solid black dendrite), the depolarisation reaches a level that allows synaptic plasticity to take place. In the example shown here, a weak context input (lemon) is paired with a strong context element (“white”) together with back-propagating dendritic depolarisation (shown by arrows). This level of depolarisation drives two kinds of plasticity—LTP at the weak, conjunctively active “lemon” synapse, and homeostatically scaling LTD at the other active context inputs (including “white”). Now, that dendritic branch will depolarise only following the conjunction of white and lemon. The collective processing of the context elements by the granule cell population will shape the feedback patterns from the commissural association system and allow refinement of the granule cell responsiveness to future contextual situations.

There is one other phenomenon which the contextual gating model can potentially explain, although we have yet to model it with our simulated grid cells, and that is conditional remapping, which is remapping to changes in one contextual stimulus that depends on what other(s) may also be present. An example of conditional remapping was given earlier in the discussion of the two-element context experiments ([13] and Figure 2), in which the response to a change in (say) color depended on the odor that was present, and vice versa. In this experiment, not only did the place cells act independently, they also acted as though they “knew” which color and odor belonged together in order to produce a given field. Theoretical considerations show that this could not occur if the context elements terminated independently on the place cell: they must be integrated somewhere upstream of the cell, possibly in its dendrites. The contextual gating model that we originally formulated [42] suggested a convergence, upstream of the place cells, between spatial inputs and contextual inputs, which is where the integration could occur, forming a combined associative input that could be thought of as “configural” (i.e., combining stimulus elements together). With the subsequent discovery of grid cells, this model was able to be given more specific detail [37]: in the updated version, the integration can occur at the place where the context elements converge, which in our model is in the dendritic tree of a dentate granule cell. By this view, context elements converging in entorhinal cortex from different sensory modalities come to coterminate on the same part of the dendritic tree (Figure 5(a)) and are able to act together to gate the same set of grid cell inputs. An illustration of how this might occur is shown in Figure 6.

### 2.2. Learning Discrete Attractor States

From the foregoing, we can see how it is that the continuous attractor dynamics in the place cell population can survive partial remapping—the attractor dynamics are actually generated in the upstream grid cells, which do not fragment when contexts are fragmented. Thus, although new place fields occur in the hybrid context states, the underlying generators—the grids—are unchanged. It is worth noting that although this scheme does not require grid cells to have remapped, they likely do also remap to even nonspatial contextual changes [45]—but whether or not they remap, the attractor hypothesis supposes that grids having the same scale will maintain their relative firing locations, thus preserving the continuous attractor dynamics.

The situation with the discrete attractors requires one more level of explanation. We have proposed that the discrete attractor states arise as a result of switching in and out of combinations of grids, but we have also noted that evidence suggests that these attractor states are learned by the dentate-CA3 network, and indeed that one function of the hippocampus might be to discover the set of attractor states that best corresponds to the states the environment can exist in. 

How can attractor states, discovered by the dentate-CA3 autoassociative network as a result of experience, act to shape the switching profiles upstream in the entorhinal-hippocampal connections? The implication is that there must be some kind of back-propagation from the dentate-CA3 system to the dendritic tree in which the proposed contextual gating occurs. It is suggested here that this back-propagation could occur within the granule cells themselves, since depolarization can travel retrogradely into the dendrites [43, 46]. Dentate gyrus neurons receive substantial back-projections from the CA3 autoassociative network via a rich set of commissural associational inputs which terminate on the proximal branches of the granule cell dendrites ([47], Figure 4(a)). Also, spikes that have been generated in granule cells are able to back-propagate into the dendrites and thus alter both the degree of depolarization and also the likelihood of synaptic plasticity there [43, 46]. An illustration of how this back-propagation might occur at the single-cell level is shown in Figure 6, in which retrograde depolarization from the commissural associational network facilitates LTP of a weak context input onto a branch of the dendritic tree, and compensating homeostatic LTD in other synapses so as to keep drive onto the cell constant. This also illustrates the process of configural context formation discussed earlier. 

The injection of network information, via the commissural-associational pathway, means that attractor states that have been formed and learned by the dentate-CA3 network can feed back into the entorhinal hippocampal connections, where the switch between patterns occurs. Thus, the attractor basins that have been formed by the dentate-CA3 network can act, in principle, to shape themselves by enhancing the connections from active incoming context inputs back in the dendrites of those same cells.

## 3. Conclusion

This paper has described continuous and discrete attractor dynamics in the hippocampal formation and proposed a mechanism for the interaction between the two attractor systems. The continuous attractor may be located in the entorhinal grid cell network, and it allows the smooth transition from one set of active place cells to the next as the animal moves around. The location of this attractor outside of the place cell network itself allows an explanation for why the continuous dynamics are preserved even when the place cells partially remap. The discrete attractor landscape arises initially from the mapping between entorhinal cortex and hippocampus, by virtue of context-mediated selection of a unique subset of grid cell afferents onto each place cell. Contextual gating can explain a number of phenomena such as rate remapping, partial remapping of place cells even when grid cells do not remap, and also conditional remapping in which the response to one contextual stimulus depends on the presence of another. By this view, the discrete attractor landscape is sculpted within the hippocampal place cell network, but the jump in state that occurs following large environmental changes arises upstream of the place cells, in the entorhinal hippocampal connections and (in situations where grid cells themselves remap) beyond. New basins in the place cell attractor landscape feed into this connectivity matrix via back-propagation of depolarization into the dendritic tree. In this way, incoming contextual inputs help the place cell network discover and learn the appropriate attractor landscape, and the resultant plasticity, in turn, shapes how the contextual inputs modulate the grid-cell/place-cell connections, and thus the place field remapping dynamics.
